# Material hardship and secure firearm storage: findings from the 2022 behavioral risk factor Surveillance System

**DOI:** 10.1186/s40621-024-00549-7

**Published:** 2024-12-19

**Authors:** Alexander Testa, Mike Henson-Garcia, Dylan B. Jackson, Karyn Fu, Kyle T. Ganson, Jason M. Nagata

**Affiliations:** 1 Department of Management, Policy and Community Health, School of Public Health, University of Texas Health Science Center at Houston, Houston, TX USA; 2 Department of Health Promotion & Behavioral Sciences, School of Public Health, University of Texas Health Science Center at Houston, Houston, TX USA; 3 Johns Hopkins Bloomberg School of Public Health, Baltimore, MD USA; 4 Rice University, Houston, TX USA; 5 Factor-Inwentash Faculty of Social Work, University of Toronto, Toronto, Canada; 6 Department of Pediatrics, University of California San Francisco, San Francisco, CA USA

**Keywords:** Firearms, Firearm Secure Storage, Material hardship, Poverty, Public Health, BRFSS

## Abstract

**Background:**

Firearm secure storage is an important public health practice due to its potential impact on reducing the incidence of accidental injuries, suicides, and thefts. Yet, there is limited research on how economic conditions might shape firearm storage patterns.

**Methods:**

This study explores the relationship between material hardship and firearm secure storage among firearm-owning households. Data from the 2022 Behavioral Risk Factor Surveillance System (BRFSS) were analyzed, including responses from 7,197 firearm-owning adults in California, Minnesota, Nevada, and New Mexico. Multinomial logistic regression models assessed the relationship between levels of material hardship and storage practices, adjusting for demographic and socioeconomic factors.

**Results:**

Among respondents, 14.3% reported firearms were stored, loaded and unlocked. Compared to respondents experiencing no hardships, those experiencing three or more material hardships incurred a 183% higher risk of storing firearms in an unsecured manner (Relative Risk Ratio = 2.828, 95% CI = 1.286, 6.220).

**Conclusion:**

This study highlights an association between greater material hardship and unsecured firearm storage. These findings emphasize the need for public health interventions that address economic barriers to safe firearm storage, potentially reducing firearm-related injuries and deaths among individuals experiencing material hardship.

**Supplementary Information:**

The online version contains supplementary material available at 10.1186/s40621-024-00549-7.

## Introduction

The United States (US) has the highest rate of gun ownership in the world [1], with approximately 300–400 million firearms in circulation [2], and 4-in-10 Americans report living in a household with a firearm [3]. Alongside the high levels of firearm ownership, the US also experiences a disproportionately high level of firearm mortality and morbidity [4], including over 48,000 firearm deaths in 2022 [5].

Firearm secure storage—i.e., storing firearms unloaded and locked—is an important public health practice due to its potential impact on reducing the incidence of accidental injuries, suicides, and thefts [6–9]. Indeed, organizations such as the American Academy of Pediatrics and the American Medical Association recommend storing firearms locked, and storing ammunition separately from the firearm [10, 11]. In addition, 27 states and Washington, DC, currently have laws requiring firearm locking [10]. 

One important correlate of firearm ownership is socioeconomic status (SES). Lower-income households are less likely to have a firearm, partly due to the high cost of firearms and ammunition [12, 13]. Even so, there is a lack of research on how household economic circumstances might influence patterns of firearm-secure storage. There are reasons to expect that economic conditions such as material hardship—i.e., the inability to pay for basic needs such as food and bills—might be relevant to how securely firearms are stored. While firearms may be obtained through various legal (i.e., purchases; gifts or inheritances) and illegal channels (i.e., thefts; illicit purchases), economic downturns that diminish household resources can affect the ability to purchase gun safes or locks needed for secure storage. Qualitative research has identified cost as a major barrier to obtaining secure gun safes with populations including firearm-owning parents and caregivers [14] and US military veterans [15]. 

In addition to financial barriers, psychological distress—often heightened in situations of material hardship—can impair decision-making and limit the capacity for precautionary actions like secure firearm storage [16]. Additionally, material hardship may be associated with lower firearm safety knowledge and/or secure storage laws, limiting awareness of recommended storage practices, or the perceived necessity of using locks and safes to prevent unauthorized access. Importantly, material hardship is often accompanied by both economic and psychological distress [17], potentially increasing the risk for suicidal ideation and/or behavior [18]. Thus, when coupled with unsecured firearm storage, firearm ownership among those experiencing greater material hardship may pose significant threats to an individual’s safety and well-being [19]. 

Given these findings, there is a strong rationale for examining how firearm storage practices in the home might vary as a function of material hardship, yet there remains a lack of research on this topic. Using a statewide sample of firearm owners in four states from the 2022 Behavioral Risk Factor Surveillance System (BRFSS), the current study investigates the relationship between material hardship and firearm secure storage among US adults. We hypothesized there would be a positive association between material hardship and unsecured firearm storage.

## Methods

Data are from the 2022 BRFSS, a nationally coordinated, state-based, continuous telephone health survey system in the US managed by the Centers for Disease Control and Prevention (CDC). The BRFSS collects data from adults aged 18 years and older residing in the United States and is designed to monitor a broad array of health-related risk behaviors. The BRFSS uses a multistage, probability-based sampling method that includes stratification and clustering to ensure coverage of diverse populations across US states and territories. Each state’s sample is weighted to adjust for differences in selection probability, nonresponse, and post-stratification factors such as gender by age group, race/ethnicity, education, marital status, tenure, gender by race/ethnicity, age group by race/ethnicity, and phone ownership [20]. 

While the survey operates in all 50 states and Washington, DC, the BRFSS includes optional state modules completed by participants residing in a subset of states on specific topics, including firearm storage and social determinants of health [20]. The analytic sample comprises 7,197 respondents who reported household firearm ownership and lived in California, Minnesota, Nevada, and New Mexico—the four states that participated in the optional modules on “Firearm Safety” and “Social Determinants and Health Equity” (refer to Appendix A for a sample selection flowchart). Across the states in the 2022 BRFSS data, Minnesota (39.3%), Nevada (38.0%), and New Mexico (37.3%) have roughly similar levels of household firearm ownership, whereas California (19.6%) has substantially lower levels.

*Unsecured firearm storage* is categorized based on responses to a series of questions detailed in Appendix B. Respondents who indicated the presence of firearms in or around the home, excluding non-functional guns, were first asked if any firearms were currently loaded (1 = yes; 0 = no). Those with loaded firearms were further asked: “Are any of these loaded firearms also unlocked?” (1 = yes; 0 = no). Based on these responses, we classified respondents into three categories: (a) firearm unloaded [reference], (b) firearm loaded and locked, and (c) firearm loaded and unlocked.

*Material hardship* is measured using a series of questions about respondents’ experiences over the past 12 months. These experiences include: (a) loss of employment, (b) receipt of Supplemental Nutrition Assistance Program (SNAP) benefits, (c) food insecurity, (d) inability to pay mortgage, rent, or utility bills, (e) threats of utility shutoff, and (f) unreliable transportation (refer to Appendix C for details). Responses to these items are summed into a single scale and categorized into four levels of hardship: 0 hardships, 1 hardship, 2 hardships, or 3 or more hardships (Kuder-Richardson Coefficient of Reliability = 0.633).

### Control variables

Control variables include respondent *age* (18–24, 25–34, 35–44, 45–54, 55–65, or 65+), *sex* (male or female), *race/ethnicity* (non-Hispanic White, non-Hispanic Black, Hispanic, or non-Hispanic other race), *marital status* (married, divorced/separated, widowed, never married, or member of an unmarried couple), *educational attainment* (less than high school, high school graduate, some college, or college graduate), *child in the home* (yes or no), a *military veteran* (yes or no), *household income* ($<25,000, $25,000 - $49,999, $50,000 - $74,999, $75,000 - $99,999, $100,000 - $149,999 or *≥* $150,000) if a respondent was told they by a healthcare professional they were *ever depressed* (yes or no), *urbanicity* (urban or rural), and *state or residence*.

### Analytic approach

Unweighted frequencies and weighted percentages were calculated. Multinomial logistic regression models were used to assess the relationship between levels of material hardship and unsecured firearm storage, adjusting for control variables listed above. Analyses were adjusted for BRFSS survey weights (*llcpwt*), primary sampling unit (*psu*), and stratification (*ststr*) information using the *svy* command in STATA v.17 (StataCorp) to weight the data to be representative of the state-level, and adjust for the complex survey design of the BRFSS. Statistical significance was determined at the *p* < .05 threshold.

## Results

Table 1 provides the weighted summary statistics and the unweighted frequencies. Among the 7,197 respondents with firearms in the household, 71.6% reported firearms stored unloaded, 14.0% reported firearms stored loaded and locked, and 14.3% reported firearms stored loaded and unlocked. Most respondents reported having zero hardships (76.8%), 14.9% reported one hardship, 3.7% reported two hardships, and 4.6% reported three or more hardships (see Table 1). Summary statistics stratified by state are provided in Appendix D.

**Fig. 1:**
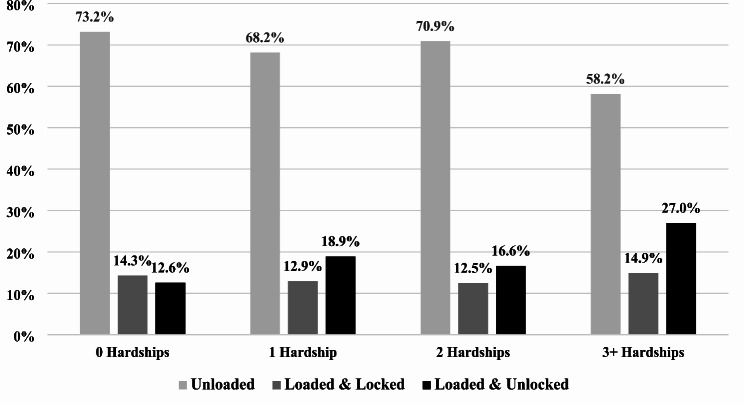
Weighted Firearm Secure Storage by Material Hardship (*N* = 7,197)

Figure 1 shows that among those with zero hardships, 73.2% stored firearms unloaded, 14.3% stored firearms loaded and locked, and 12.6% stored firearms loaded and unlocked. In comparison, among those with three or more hardships, 58.2% stored firearms unloaded, 14.9% stored firearms loaded and locked, and 27.0% stored firearms loaded and unlocked. 

**Table 1:** Summary statistics (*N* = 7,197)

Variables	Unweighted Frequency	Weighted %
*Firearm Storage*		
Unloaded	5,346	71.6%
Loaded & Locked	870	14.0%
Loaded & Unlocked	981	14.3%
*Material Hardship*		
0	5,971	76.8%
1	794	14.9%
2	226	3.7%
3+	206	4.7%
*Age*		
18–24	282	7.3%
25–34	650	16.6%
35–44	992	17.2%
45–54	1,128	14.6%
55–64	1,598	18.8%
65+	2,547	25.4%
*Sex*		
Female	3,050	40.5%
Male	4,147	59.5%
*Race/Ethnicity*		
Non-Hispanic White	5,940	57.8%
Non-Hispanic Black	160	5.7%
Hispanic	707	22.5%
Non-Hispanic Other Race	390	14.0%
*Marital Status*		
Married	4,549	60.1%
Divorced/Separated	895	10.3%
Widowed	532	4.1%
Never married	913	19.5%
Member of an unmarried couple	308	5.9%
*Educational Attainment*		
Less than High School	155	6.5%
High School Graduate	1,410	24.7%
Some College	2,273	36.9%
College Graduate	3,359	31.9%
*Child in Home*		
No	5,342	64.8%
Yes	1,855	35.2%
*Military Veteran*		
No	6,018	83.8%
Yes	1,179	16.2%
*Household Income*		
Less than $25,000	490	7.3%
$25,000 - $49,999	1,472	19.6%
$50,000 - $74,999	1,307	14.1%
$75,000 - $99,999	1,206	15.2%
$100,000 - $149,999	1,426	22.0%
$150,000 or more	1,296	21.8%
*Ever Told Had Depression*		
No	5,821	82.3%
Yes	1,376	17.7%
*Urbanicity*		
Rural	881	6.0%
Urban	6,316	94.0%
*State of Residence*		
California	1,094	60.5%
Minnesota	4,109	21.2%
Nevada	763	10.5%
New Mexico	1,231	7.8%

Table 2 reports the results of the multinomial logistic regression analysis with the reference categories set to respondents who report having no hardships and those storing firearms unloaded. The results show that, including control variables, respondents who reported three or more hardships had a 2.8 times higher risk of storing firearms loaded and unlocked than those with no hardship (Relative Risk Ratio [RRR] = 2.828, 95% Confidence Interval [CI] = 1.286, 6.220, *p* = .010). No other level of material hardship was significantly associated with the outcome. In addition, there is no significant association between household income and firearm secure storage (results not shown).

**Table 2:** Results of Multinomial Logistic regression of Firearm Storage on Material Hardship from the 2022 BRFSS (*N* = 7,197)

Variables	Loaded & Locked vs. Unloaded		Loaded & Unlocked vs. Unloaded	
	RRR	95% CI	RRR	95% CI
0 Hardships (Reference)	–	–	–	–
1 Hardship	0.907	(0.575–1.433)	1.743	(0.993–3.061)
2 Hardships	0.702	(0.338–1.458)	1.173	(0.579–2.376)
3 + Hardships	1.288	(0.622–2.663)	2.828**	(1.286–6.220)

*** p<0.001, ** p<0.01, * p<0.05

RRR = relative risk ratio; CI = confidence interval

Control variables include: respondent age, sex, race/ethnicity, marital status, educational attainment, child in home, military veteran, household income, ever depressed, urbanicity, and state of residence

Standard errors are calculated using survey weights

Appendix E provides six separate multinomial regression analyses of each type of material hardship on firearm safe storage. The results document statistically significant associations between storing firearms loaded and unlocked among respondents reporting food insecurity (RRR = 4.979, CI = 2.043, 12.133, *p* < .001), difficulty paying bills (RRR = 1.928, CI = 1.058, 3.516, *p* = .032), having utilities threatened to be shut off (RRR = 2.922, CI = 1.221, 6.992, *p* = .016), and transportation difficulties (RRR = 2.435, CI = 1.198, 4.947, *p* = .014)

### Supplemental analyses

The regression analysis in Table 2 included household income and material hardship, which were moderately correlated (*r* = .280). Supplemental analyses indicate that when removing household income as a covariate, reporting three or more hardships retains a similar 2.7 times higher risk of storing firearms loaded and unlocked (RRR = 2.774, 95% CI = 1.183, 6.503, *p* = .019). When removing material hardship from the regression model, there is no statistically significant association between household income and firearm secure storage. These results suggest that material hardship, rather than overall household income levels, are uniquely associated with firearm secure storage (see Appendix F).

## Discussion

The analysis of data from more than 7,000 US adults living in firearm-owning households provides new evidence that those experiencing material hardship are significantly more likely to store their firearms loaded and unlocked. These findings are consistent with prior qualitative research that has found cost to be a barrier for firearm secure storage among firearm-owning parents and caregivers [14] and US military veterans [15]. Considering that the US Surgeon General has declared firearm morbidity and mortality to be a serious public health crisis [21], and that both material hardship [18] and unsecured firearm storage [22, 23] are risk factors for suicide—and those risks may compound when they co-occur—the findings raise implications for public health.

Current policies and initiatives promoting firearm secure storage, such as laws requiring firearm locking implemented by a majority of states [10] and recent executive actions promoted by the Biden-Harris administration to promote safe storage of firearms, often overlook the impact of economic constraints on firearm storage practices [24]. Tailored interventions could address these gaps, including financial assistance for purchasing safety devices like gun locks and safes, subsidized firearm safety education, and integration of financial assistance programs with firearm safety initiatives. In addition, programs that link financial assistance to hospital-based firearm safety initiatives could leverage healthcare settings to promote effective gun safety education and intervention strategies. By addressing the underlying economic factors contributing to unsafe storage practices, such measures could significantly reduce preventable firearm-related injuries and deaths in vulnerable populations. Additionally, focused efforts should include education on the benefits of safe firearm storage, paired with the distribution of locking devices, as evidence shows that safe storage education is most effective when accompanied by access to secure storage options [7]. 

This study has several limitations that can be expanded upon in future research. Only four states in the BRFSS have data on firearm storage practices and material hardship from optional state modules, limiting the findings’ generalizability to the broader US population. Notably, all four states in the sample currently have laws in place requiring the locking of firearms [10]. Thus, how the findings of this study generalize in states without such laws is an important question for future inquiry. Additionally, the study’s cross-sectional nature prevents drawing causal inferences from the data. In particular, we cannot discern when a household acquired a firearm, the dynamics of when material hardship began or ended, and if a household transitioned away from secured storage behavior after material hardship took place. The skip pattern used to inquire about how a firearm was stored in the BRFSS also prohibits an analysis of whether a firearm was stored unlocked, or whether the firearm was loaded or not. Furthermore, the BRFSS’s reliance on self-reported information may be subject to recall bias. Future research should expand to include more states and employ longitudinal designs to understand better the temporal dynamics between material hardship and firearm storage practices. On this point, it would be valuable for future research to collect more granular detail on objective and subjective measures of economic hardship and distress, as well as potential mediating mechanisms such as psychosocial distress, knowledge of firearm secure storage benefits, and budget constraints that inhibit the purchasing of secure storage devices.

## Conclusions

The current study is the first to our knowledge to establish a link between material hardship and firearm secure storage. Further data collection and research are imperative to deepen an understanding of how material hardship influences firearm-related behaviors. Such knowledge is crucial for developing targeted public health interventions that can reduce preventable firearm-related injuries and deaths while addressing the root causes of unsafe firearm storage practices linked to economic difficulties.

## Electronic supplementary material

Supplementary Material 1

## Data Availability

The datasets generated during and/or analyzed during the current study are available from the corresponding author upon reasonable request. The BRFSS data are publicly available at: https://www.cdc.gov/brfss/index.htmlThe datasets generated during and/or analyzed during the current study are available from the corresponding author upon reasonable request. The BRFSS data are publicly available at: https://www.cdc.gov/brfss/index.html.
