# Supplementary material for: Material hardship and secure firearm storage: findings from the 2022 behavioral risk factor Surveillance System
Source: Inj Epidemiol. 2024 Dec 19;11:69. doi: 10.1186/s40621-024-00549-7 (PMC11656984; doi:10.1186/s40621-024-00549-7)
Supplement: Supplementary file 1 — Supplementary Material 1 [file 40621_2024_549_MOESM1_ESM.docx]

**Appendix A: Sample Selection Flowchart**

| Participated in 2022 BRFSS | *N* = 445,132  (54 States & Territories) |
| --- | --- |
|  | **↓** |
| Participated in the “Social Determinants and Health Equity” Module | *N* = 253,790  (15 States and Territories) |
|  | **↓** |
| Participated in “Firearm Safety Module” | *N* = 39,800  (5 States) |
|  | **↓** |
| The Respondent Household Owns a Firearm | *N* = 13,839  (5 States) |
|  | **↓** |
| Answered Firearm Safe Storage Questions | *N* = 13,399  (5 States) |
|  | **↓** |
| Answered Questions on Material Hardship | *N* = 8,445  (4 States) |
| Has Data on Control Variables | ↓ |
|  | *N* = 7,197  (4 States) |

**Appendix B: Firearm Safety Module**

| **Prologue:** The next questions are about safety and firearms. Some people keep guns for recreational purposes such as hunting or sport shooting. People also keep guns in the home for protection. Please include firearms such as pistols, revolvers, shotguns, and rifles; but not BB guns or guns that cannot fire. Include those kept in a garage, outdoor storage area, or motor vehicle. | | | | |
| --- | --- | --- | --- | --- |
| **Question text** | **Responses** | **Skip Info** | **Interviewer Notes** |  |
| Are any firearms now kept in or around your home? | Yes | Answer the next firearm question | Do not include guns that cannot fire; include those kept in cars, or outdoor storage. |  |
|  | No  Don’t know/not sure  Refused | Skip next firearm questions |  |  |
| Are any of these firearms now loaded? | Yes | Answer the next firearm question |  |  |
|  | No  Don’t know/not sure  Refused | Skip next firearm questions |  |  |
| Are any of these loaded firearms also unlocked? | Yes  No  Don’t know/not sure  Refused |  | By unlocked, we mean you do not need a key or a combination or a hand/fingerprint to get the gun or to fire it. Don’t count the safety as a lock. |  |

**Appendix C: Material Hardship Measures**

| **Material Hardship**  **Item** | **Question** | **Original Coding** | **Coding for Analysis** | **Prevalence** |
| --- | --- | --- | --- | --- |
| Employment Hardship | In the past 12 months have you lost employment or had hours reduced? | 1 Yes  2 No  7 Don’t Know/Not Sure  9 Refused | 0 No  1 Yes  *Don’t Know/Not Sure or Refused recoded as missing | 11.1% |
| Public Assistance | During the past 12 months, have you received food stamps, also called SNAP, the Supplemental Nutrition Assistance Program on an EBT card? | 1 Yes  2 No  7 Don’t Know/Not Sure  9 Refused | 0 No  1 Yes  *Don’t Know/Not Sure or Refused recoded as missing | 7.2% |
| Food Insecurity | During the past 12 months how often did the food that you bought not last, and you didn’t have money to get more? Was that… | 1 Always  2 Usually  3 Sometimes  4 Rarely  5 Never  7 Don’t Know/Not Sure  9 Refused | 0 Never, Rarely, or Sometimes  1 Always or Usually  *Don’t Know/Not Sure or Refused recoded as missing | 3.4% |
| Pay Bills | During the last 12 months, was there a time when you were not able to pay your mortgage, rent or utility bills? | 1 Yes  2 No  7 Don’t Know/Not Sure  9 Refused | 0 No  1 Yes  *Don’t Know/Not Sure or Refused recoded as missing | 7.9% |
| Utilities Shut Off | During the last 12 months was there a time when an electric, gas, oil, or water company threatened to shut off services? | 1 Yes  2 No  7 Don’t Know/Not Sure  9 Refused | 0 No or not Married  1 Yes  *Don’t Know/Not Sure or Refused recoded as missing | 6.5% |
| Transportation Difficulties | During the past 12 months has a lack of reliable transportation kept you from medical appointments, meetings, work, or from getting things needed for daily living? | 1 Yes  2 No  7 Don’t Know/Not Sure  9 Refused | 0 No or not Married  1 Yes  *Don’t Know/Not Sure or Refused recoded as missing | 4.9% |

**Appendix D: Summary Statistics Stratified by State (*N* = 7,197)**

| **Variables** | **California**  **(N = 1,094)** | **Minnesota**  **(N = 4,109)** | **Nevada**  **(N = 763)** | **New Mexico**  **(N = 1,231)** | ***p*-value^a^** |
| --- | --- | --- | --- | --- | --- |
| *Firearm Storage* |  |  |  |  | <0.001 |
| Unloaded | 72.9% (802) | 79.6% (3,359) | 58.8% (447) | 57.6% (738) |  |
| Loaded & Locked | 14.0% (164) | 9.2% (344) | 18.8% (140) | 20.7% (222) |  |
| Loaded & Unlocked | 13.1% (128) | 11.2% (406) | 22.3% (176) | 21.7% (271) |  |
| *Material Hardship* |  |  |  |  | <0.001 |
| 0 | 75.6% (868) | 83.5% (3,549) | 74.2% (608) | 71.3% (946) |  |
| 1 | 15.6% (145) | 11.1% (381) | 16.1% (99) | 17.6% (169) |  |
| 2 | 3.3% (38) | 2.8% (100) | 6.5% (33) | 5.0% (55) |  |
| 3+ | 5.5% (43) | 2.6% (79) | 3.1% (23) | 6.0% (61) |  |
| *Age* |  |  |  |  | 0.066 |
| 18-24 | 6.8% (51) | 7.6% (152) | 7.4% (34) | 10.0% (45) |  |
| 25-34 | 17.2% (137) | 14.1% (339) | 19.8% (78) | 14.6% (96) |  |
| 35-44 | 17.4% (155) | 16.9% (591) | 18.2% (101) | 15.5% (145) |  |
| 45-54 | 13.0% (137) | 16.5% (693) | 19.2% (126) | 15.8% (172) |  |
| 55-64 | 19.4% (235) | 20.0% (956) | 14.0% (146) | 17.8% (261) |  |
| 65+ | 26.2% (379) | 24.9% (1,378) | 21.4% (278) | 26.4% (512) |  |
| *Sex* |  |  |  |  | 0.370 |
| Female | 39.4% (412) | 41.6% (1,701) | 44.2% (344) | 40.9% (593) |  |
| Male | 60.6% (682) | 58.4% (2,408) | 55.8% (419) | 59.1% (638) |  |
| *Race/Ethnicity* |  |  |  |  | <0.001 |
| Non-Hispanic White | 48.1% (721) | 89.1% (3,849) | 59.5% (612) | 45.4% (758) |  |
| Non-Hispanic Black | 6.8% (65) | 2.8% (53) | 7.5% (28) | 2.1% (14) |  |
| Hispanic | 28.3% (201) | 2.1% (74) | 16.1% (65) | 42.0% (367) |  |
| Non-Hispanic Other Race | 16.9% (107) | 5.9% (133) | 16.8% (58) | 10.4% (92) |  |
| *Marital Status* |  |  |  |  | <0.001 |
| Married | 59.3% (619) | 66.4% (2,781) | 56.9% (431) | 53.9% (718) |  |
| Divorced/Separated | 9.5% (145) | 9.8% (462) | 13.1% (120) | 14.5% (168) |  |
| Widowed | 3.4% (70) | 4.4% (239) | 5.5% (68) | 7.3% (155) |  |
| Never married | 21.2% (195) | 15.7% (495) | 19.5% (103) | 15.8% (120) |  |
| Member of an unmarried couple | 6.6% (65) | 3.6% (132) | 5.0% (41) | 8.5% (70) |  |
| *Educational Attainment* |  |  |  |  | 0.005 |
| Less than High School | 8.1% (28) | 4.1% (65) | 3.3% (15) | 5.7% (47) |  |
| High School Graduate | 24.5% (200) | 23.6% (769) | 26.3% (158) | 26.5% (283) |  |
| Some College | 34.6% (303) | 39.7% (1,353) | 43.6% (255) | 38.6% (362) |  |
| College Graduate | 32.9% (563) | 32.6% (1,922) | 26.9% (335) | 29.2% (539) |  |
| *Child in Home* |  |  |  |  | 0.663 |
| No | 64.7% (808) | 66.1% (3,043) | 62.3% (565) | 65.2% (926) |  |
| Yes | 35.3% (286) | 33.9% (1,066) | 37.7% (198) | 34.8% (305) |  |
| *Military Veteran* |  |  |  |  | 0.133 |
| No | 83.3% (901) | 86.7% (3,505) | 81.9% (620) | 82.0% (992) |  |
| Yes | 16.7% (193) | 13.3% (604) | 18.1% (143) | 18.0% (239) |  |
| *Household Income* |  |  |  |  | <0.001 |
| Less than $25,000 | 7.5% (78) | 5.2% (217) | 7.7% (60) | 10.4% (135) |  |
| $25,000 - $49,999 | 19.6% (194) | 17.7% (771) | 15.5% (143) | 30.2% (364) |  |
| $50,000 - $74,999 | 11.2% (140) | 19.8% (797) | 17.3% (130) | 17.0% (240) |  |
| $75,000 - $99,999 | 13.0% (146) | 18.1% (712) | 19.7% (142) | 17.9% (206) |  |
| $100,000 - $149,999 | 23.9% (256) | 21.1% (859) | 19.8% (156) | 12.5% (155) |  |
| $150,000 or more | 24.7% (280) | 18.0% (753) | 19.9% (132) | 12.0% (131) |  |
| *Ever Told Had Depression* |  |  |  |  | 0.074 |
| No | 83.9% (900) | 79.5% (3,292) | 80.0% (629) | 81.1% (1,000) |  |
| Yes | 16.1% (194) | 20.5% (817) | 20.0% (134) | 18.9% (231) |  |
| *Urbanicity* |  |  |  |  | <0.001 |
| Rural | 3.2% (30) | 15.9% (709) | 2.4% (36) | 5.7% (106) |  |
| Urban | 96.8% (1,064) | 84.1% (3,400) | 97.6% (727) | 94.3% (1,125) |  |

^a^p-value represents the results from Chi-Squared tests. The table presents weighted percentages (unweighted frequencies)

**Appendix E: Results of Multinomial Logistic Regression of Firearm Storage on Type of Material Hardship (*N* = 7,197)**

|  | **Loaded & Locked vs. Unloaded** | | **Loaded & Unlocked vs. Unloaded** | |
| --- | --- | --- | --- | --- |
| **Variables** | **RRR** | **95% CI** | **RRR** | **95% CI** |
| **Type of Material Hardship** |  |  |  |  |
| Employment Hardship | 1.056 | (0.634 - 1.758) | 1.527 | (0.952 - 2.450) |
| Public Assistance | 1.270 | (0.726 - 2.223) | 1.491 | (0.798 - 2.787) |
| Food Insecurity | 1.070 | (0.447 - 2.560) | 4.979*** | (2.043 - 12.133) |
| Difficulty Paying Bills | 0.844 | (0.489 - 1.455) | 1.928* | (1.058 - 3.516) |
| Utilities Shut Off | 1.125 | (0.592 - 2.136) | 2.922* | (1.221 - 6.992) |
| Transportation Difficulties | 1.292 | (0.650 - 2.568) | 2.435* | (1.198 - 4.947) |

*** p<0.001, ** p<0.01, * p<0.05

*Abbreviations*: RRR = relative risk ratio; CI = confidence interval

Control variables include: respondent age, sex, race/ethnicity, marital status, educational attainment, child in home, military veteran, household income, ever depressed, self- urbanicity, and state of residence.

**Appendix F: Results of Multinomial Logistic Regression of Firearm Storage on Material Hardship and Household Income (*N* = 7,197)**

|  | **Loaded & Locked vs. Unloaded** | | **Loaded & Unlocked vs. Unloaded** | |
| --- | --- | --- | --- | --- |
| **Variables** | **RRR** | **95% CI** | **RRR** | **95% CI** |
| **Panel A: Material Hardship** |  |  |  |  |
| 0 Hardships (Reference) | — | — | — | — |
| 1 Hardship | 0.897 | (0.562 - 1.432) | 1.682 | (0.965 - 2.932) |
| 2 Hardships | 0.681 | (0.332 - 1.398) | 1.131 | (0.549 - 2.328) |
| 3+ Hardships | 1.228 | (0.589 - 2.561) | 2.774* | (1.183 - 6.503) |
| **Panel B: Household Income** |  |  |  |  |
| Less than $25,000 (Reference) | — | — | — | — |
| $25,000 - $49,999 | 1.206 | (0.654 - 2.223) | 0.569 | (0.285 - 1.134) |
| $50,000 - $74,999 | 0.926 | (0.503 - 1.704) | 0.588 | (0.283 - 1.219) |
| $75,000 - $99,999 | 1.393 | (0.655 - 2.961) | 0.658 | (0.325 - 1.332) |
| $100,000 - $149,999 | 1.070 | (0.558 - 2.051) | 0.727 | (0.331 - 1.598) |
| $150,000 or more | 1.156 | (0.593 - 2.252) | 0.847 | (0.404 - 1.777) |

*** p<0.001, ** p<0.01, * p<0.05

*Abbreviations*: RRR = relative risk ratio; CI = confidence interval

Control variables include: respondent age, sex, race/ethnicity, marital status, educational attainment, child in home, military veteran, ever depressed, urbanicity, and state of residence.
